# Supplementary material for: Patterns of Intron Gain and Loss in Fungi
Source: PLoS Biol. 2004 Nov 30;2(12):e422. doi: 10.1371/journal.pbio.0020422 (PMC532390; doi:10.1371/journal.pbio.0020422)
Supplement: Table S1 — Also available at http://genes.mit.edu/NielsenEtAl/. (4.3 MB ZIP). [file pbio.0020422.st001.zip › NielsenEtAl/html/187.html]

AN6299.1.NCU04457.1.MG08036.1.FG09673.1


```
 CLUSTAL W (1.82) Multiple Sequence Alignments - Introns Inserted


Sequence 1: MG08036.1	757 aa
Sequence 2: FG09673.1	623 aa
Sequence 3: NCU04457.1	798 aa
Sequence 4: AN6299.1	691 aa
Alignment Length: 827 aa
Number Identitical Residues: 245 aa
Alignment Score (without introns) 12088


MG08036.1 	MDATTEAMATAHELYARSGGIGGGDAASRPPIFKLVGIILAISSGAFIGTSFVLKKYGLL
NCU04457.1	MDQAAQILEVAHHVYARADDAAD-LHATRPPSYKIIGILLAVGSGFFIGTSFVVKKMGLL
FG09673.1 	MDA---------DIFARSGGIGG-DTSDRPGFYKPVGIGLAVGSGCFIGVSFVLKKFGLL
AN6299.1  	-----------MDLSPRSGISSG---GDRPPAYKAIGISLAVASGVFIGVSFVLKKVGLL
          	            .: .*:.  ..   . **  :* :** **:.** ***.***:** ***

MG08036.1 	KANEKYNEEPGEGYGYLKNFWWWTGMILMIIGEICNFVAYAFTDAILVTPLGALSVVITA
NCU04457.1	KANEKYNEVAGEGYGYLKNAWWWAGMILMLIGELLNFAAYMFVDAILVTPLGALSVVVAT
FG09673.1 	RANEKYNEVAGEGYGYLKNFWWWTGMILMIIGEILNFVAYAFTDAILVTPLGALSVVIAA
AN6299.1  	RANVKYNEEAGEGYGYLKNLWWWSGMTLMIIGEICNFVAYAFVDAILVTPLGALSVVITT
          	:** **** .********* ***:** **:***: **.** *.**************:::

MG08036.1 	ILSAIFLKERLSLVGKVSCFLCIVGSVVIVMNGPQESSVATIEQMQSFVIHPAFLSYTGV
NCU04457.1	VGSAIVLKERLSMIGKVSCFLCIVGSVVIVLNAPQESAVANIQQFQQFVVTPSFLSYAGV
FG09673.1 	ILSSIFLKERLSLVGKVGCFLCIVGSVVIVMNAPHSSSVSDIQQMQGYFITPGFLTYAGI
AN6299.1  	ILSAIFLKERLSFVGKVGCFSCILGSVVIALNAPEQSSVADIQDMKKYVIAPGFLSYAGV
          	: *:*.******::***.** **:*****.:*.*..*:*: *:::: :.: *.**:*:*:

MG08036.1 	IVIGSAITAFYCGPRWGKKNMLVYISICSWIGGLSVVATQGLGAAIVTQIGGKPQFNQWF
NCU04457.1	IVLGAVIAAWYAGPRWGNKNMLVYISICSWIGGLSVVSTQGLGSAIVAQAGGEAQFKGWF
FG09673.1 	ILVGSAVTAIWVAPKYGNKNMLVYISICSWIGGLSVASTQGLGAAIIAWIGGKPQYKEWF
AN6299.1  	IIVACAITAIWAGPRYGKRSMFVYISICSLIGGLSVVATQGLGAALLAQINGEAQFKEWF
          	*::...::* : .*::*::.*:******* ******.:*****:*:::  .*:.*:: **

MG08036.1 	TYVLLAFVIATLLTEIIFLN0KALNLFNAAMVTPTYYVYFTSTTIITSTVLFRGFKGTPT
NCU04457.1	IYIVIIFFIASLLTELIYLN0KALNLFNAAMVTPTYYVYFTSTTIITSAVLFKGFKGTAV
FG09673.1 	LWVLFVFVIGTLLTEIIFLN0KALNIYNAALVTPTYYVYFTSTTIITSAILYQGFKGSVQ
AN6299.1  	MYVLLVFVVATLLTEIIYLN0KALNIFNAALVTPTYYVFFTSSTIISSAILFRGFKGTGT
          	 :::: *.:.:****:*:** ****::***:*******:***:***:*::*::****:  

MG08036.1 	SIITVVLGFLTICAGVVLLQLSKSAKDVPDTAVFNGDLDQIHTIAEQEQPETEPKADAIR
NCU04457.1	SIVTVVFGFLTICSGVVLLQLSKSAKDVPDAAVLSGDLDQIRTVAEQEQPETEPKADALR
FG09673.1 	SIVTVVLGFLTICSGVVLLQISKSAKDVPDSAVFNGDLDQIQTIVEQEQPETDPKADAIR
AN6299.1  	QIASVILGFLQICAGVVLLQLSKSAKDVPDAAVFKGDLDQVREVATQEEPEFEPKADAIR
          	.* :*::*** **:******:*********:**:.*****:: :. **:** :*****:*

MG08036.1 	GTAAIVRRLSSAR-MRMEAEELKRLHEEKQ-AETLDPVNED-AMAQYEWDGLRRRRT---
NCU04457.1	GAAAIVRRLSSAKQQKMEMEELKRLHEEKM-REALETVGED--GPVYEWDGLRRRKTVLG
FG09673.1 	GTAAIVRQMSQRR-LRMEAEELKRLHEEKMMMETMDPIREDGSQPEWEWDGLRRRRTMTF
AN6299.1  	GTAAIIRRLSTPR-RNVEAEEARRYLRERQ-EDQLRPPADN---EIVEWDGLRRRKT---
          	*:***:*::*  :  .:* ** :*  .*:   : : .  ::      ********:*   

MG08036.1 	---TYGSRRSRAMTSPNP--------FVIPPPTP--HPPLGWSHFPTQEELDALE-----
NCU04457.1	--SNSASQRSRAATSAT---------FQLPMPTP--HPPLGWSHFPTEEELAAAA-----
FG09673.1 	RTGTMSSSRAGSVTSPPPQVPPISPRFAVRPSSPRQHPPLGMSQFPPIDDDDDNDNDGNN
AN6299.1  	----VLSEKSQR-----------------RTPTP--LPPLGMSRFPDDLSEDSHN-----
          	      * ::                     .:*   **** *:**   .          

MG08036.1 	RPSTPGAMSSIVGTIRSRARSIMVKDGDTPARHSARPGLESIHSPMHPVQLTEVNLQQTG
NCU04457.1	RPASP-ALSSIVGTIRNRARSVLLPGHPNFRPTSASN---KVQSPMHPVQLTEIAVPAQD
FG09673.1 	RQETNTLFSSIAGTIRGRARGFTNFSG-RHANDDHEDDKPRPRSPLHPVPLTEIAVPRDK
AN6299.1  	---TSPSVHTFMNEVRSRTSSIMS----------------SPWRPIPGDQQTHGPVLEPD
          	   :   . :: . :*.*: ..                      *:     *.  :    

MG08036.1 	QDLEAGLYPE------KKDGKYYQ--GSTLSVPGTSRSAPAGRHRGPSNASDLSSLGPPP
NCU04457.1	FDEETGYYPPGSAAGPSSKGYMYDRPGSRGSISSSARRVQFSTDANHPSSSTLAAPLAPP
FG09673.1 	ADEEHAAYYG------QTREHTFN--GQDTEYRGASFRS----------GNSLAPPTPPP
AN6299.1  	PKVVDTSYPG-------------QHPPRAVSWVDEEQDQ-----------SHLAAPAPPP
          	 .     *               : .    .  .                . *:.  .**

MG08036.1 	TPPPHGGARRQFSFQKMGFGLGMRGSGASQHQASPSPGGFGSDGIEEARPRTGRGYS---
NCU04457.1	PLRTPTGATRQFSFQSV-FKRGDRRSQHTHTQSDGANSHKSSHSSDKQDRDKLTTHRGLS
FG09673.1 	HGQPPDITRRQFSFHNV-FRRPNSSSRSTSHVEDP-------------------------
AN6299.1  	H--------RHFSFQKI-FSRAKSPVDPSSADSRSHTRSRSPRGILRRG-----------
          	         *:***:.: *         :           .. .                

MG08036.1 	-SPITKGASEEERVGLVTNDVKSLPTLPRYDEVSEENELLPEEGGAYSDESRRTP--PRA
NCU04457.1	TPHIRRGASEEETLGLVRGDSRDFRTTPRTSKHYDDDD--GDDGSEEFEEVGRYMDEKQS
FG09673.1 	--------TEEEQAGLVKDDSR--------------------PGPAYH------------
AN6299.1  	--HMRNMTSEEEHLGLVQGDKVSGYTNEDEKHERTRTN---IQSNHDEDDYETYNYDTYD
          	       ::***  *** .*  .  :    .      .     .    ..      .   

MG08036.1 	KYGRGITESPPRAR--------------QTLEEAEEASLAEYEASRKRFNDTRAASRDRS
NCU04457.1	RYGPSITHSPPRAPGVTGVRGGTEQQYPGGEEEDVKKEYTEHMESGGQGQSQGQGQQHRS
FG09673.1 	------------------------------------------------------------
AN6299.1  	YYDDGKLDDLPSGRRLDSHSSLYPPRLRLNPLSPLPDNESALDVSHIRNDQQQYQPQPEQ
          	  . .   . . .    .  .           .    . :    :    ..        .

MG08036.1 	GDP------------------GSSRDRGSPPARQSRRPPPGGNNGAFI
NCU04457.1	SRSDINSYRERLRRIREHERHSDNNGSGSGPSSPSKSSSNRGDGGGFI
FG09673.1 	------------------------------------------------
AN6299.1  	QQP----------------LESSKGSLLTIPRRQSGWRRQDSASSG--
          	  .                  ... .  : .   :      . ...
```
